# A Novel Strategy for Quantitative Analysis of Major Ginsenosides in Panacis Japonici Rhizoma with a Standardized Reference Fraction

**DOI:** 10.3390/molecules22122067

**Published:** 2017-11-27

**Authors:** Fan-Cheng Meng, Qiu-Shuang Wu, Ruibing Wang, Shao-Ping Li, Li-Gen Lin, Ping Chen, Qing-Wen Zhang

**Affiliations:** 1 State Key Laboratory of Quality Research in Chinese Medicine, Institute of Chinese Medical Sciences, University of Macau, Macao 999078, China; yb37534@umac.mo (F.-C.M.); wuqiushuang1991@126.com (Q.-S.W.); rwang@umac.mo (R.W.); spli@umac.mo (S.-P.L.); LigenL@umac.mo (L.-G.L); 2 College of Biology and Pharmaceutical engineering, Wuhan Polytechnic University, Wuhan 430023, China; chenpingvip24@163.com

**Keywords:** *Panax japonicus* C. A. Mey., Panacis Japonici Rhizoma, triterpenoid saponin, ginsenoside, chikusetsusaponin, pseudoginsenoside, standardized reference fraction, quantification, quality control, HPLC

## Abstract

Panacis Japonici Rhizoma (Zhu-Jie-Shen in Chinese), the root of *P. japonicus* C.A. Mey., is commonly used in traditional Chinese Medicine. Saponins are the major bioactive compounds in this herb. The similarity of polarity and structure of the natural products in herb caused the difficulty of purification and resulted in the shortage and high cost of the reference compounds, which has greatly hindered efforts toward quantification in quality control. A novel strategy using a standardized reference fraction for qualification of the major saponins in Panacis Japonici Rhizoma was proposed to easily and effectively control the quality of PJR. The strategy is feasible and reliable, and the methodology of the developed approach is also validated. The standardized reference fraction was used for quantification, which might solve the shortage of the pure reference compounds in the quality control of herbal medicines.

## 1. Introduction

Most plants in the *Panax* genus have been used as traditional Chinese medicines for different medical purposes for a long time. Ginseng Radix et Rhizoma (GRR Asia Ginseng, the root and rhizome of *Panax ginseng* C.A. Mey.), Notoginseng Radix et Rhizoma (NRR, San-Qi, the root and rhizome of *P. notoginseng* F.H. Chen), Panacis Quinquefolii Radix (PQR, American ginseng, the root of *P. quinquefolium* L.), and Panacis Japonici Rhizoma (PJR, Zhu-Jie-Shen, the root of *P. japonicus* C.A. Mey.) were officially documented in Chinese Pharmacopeia [1]. Ginsenosides (dammarane or oleanane-type saponins) are considered as the major bioactive ingredients. The types and contents of ginsenosides vary greatly in different *Panax* species. Both GRR and PQR contain high contents of dammarane-type saponins, as well as certain amounts of oleanane-type saponins, which are used as medicinal tonic. NRR contains much higher content of dammarane-type saponins, but no oleanane-type saponin was isolated in pure form even though oleanane-type saponins were found in a pretty low content in NRR. NRR is used for promoting blood circulation. PJR contains both dammarane and oleanane-type saponins, and the content of oleanane-type saponins is about ten times higher than that of dammarane-type saponins. PJR is used as an analgesic, an anti-cough medicine and as expectorant [1,2,3,4,5,6,7]. The total and individual saponins from the genus *Panax* have been found to have multiple pharmacological activities such as anti-cancer, anti-inflammatory, anti-diabetic, cardioprotective, and neuroprotective activities [8,9,10]. Quantification of multiple ginsenosides is a rational strategy for quality control for those herbs and their products [11,12,13,14].

Reference compounds play a key role in quality control of traditional Chinese Medicine. However, high-purity reference compounds from herbal medicines are very expensive and usually difficult to obtain. Herbs may contain many other compounds with the polarity and structure similar to the selected reference compounds, which may have prevented the large scale preparative separation of these reference compounds. The shortage and high cost of the reference compounds has greatly hindered the implementation of multi-components quantification in routine quality control. The quantitative analysis of multi-components with single-marker strategy (QAMS) has been developed and can partially lessen the bottleneck effect of missing reference compounds [15,16]. However, QAMS still requires high-purity reference compounds and has the challenge of the peak identification using relative retention time [17].

**Figure 1:**
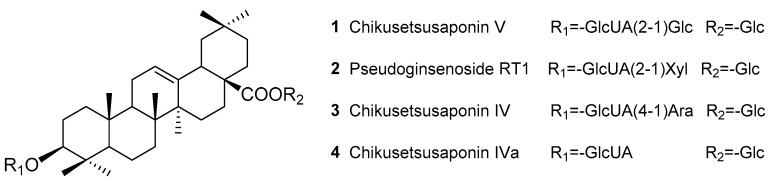
Chemical structures of analytes **1**–**4**.

In this study, a convenient and effective novel strategy using the total *P. japonicus* saponins (TPJS) as the standardized reference fraction for qualification of the major saponins, chikusetsusaponin IV (CS-IV), chikusetsusaponin IVa (CS-IVa), chikusetsusaponin V (CS-V), and pseudoginsenoside RT1 (PG-RT1) (Figure 1) in PJR was developed.

## 2. Results

### 2.1. Preparation of TPJS

Macroporous resins have been found to be an efficient material to enrich ginsenosides from *Panax* spp. [13,18,19,20,21,22]. The D101 macroporous resin was selected for the separation of TPJS according to previous studies [18,19]. Preliminary investigation found that the saponins in *P. japonicus* absorbed in D101 macroporous resin could not be desorbed by aqueous ethanol under 20% (*v*/*v*) and the complete desorption could be achieved by 80% aqueous ethanol (*v*/*v*). Thus, water and 20% aqueous ethanol (*v*/*v*) were selected for removing impurity and 80% aqueous ethanol (*v*/*v*) was selected to elute the total saponin. The PJR (1 kg) was extracted by 95% ethanol (*v*/*v*) for three times (5 L each time). The pooled extracts were dried in vacuum and then suspended in water. The suspension was then loaded to a D101 macroporous resin (1.0 kg) column and eluted with water, 20% aqueous ethanol (*v*/*v*), and 80% aqueous ethanol (*v*/*v*), respectively. The 80% aqueous ethanol (*v*/*v*) fraction was collected and dried to afford TPJS 106.5 g. The separation of total saponins on D101 macroporous column could be easily scaled up, even to a kilogram scale, which is more productive than the separation of individual pure saponin, usually at the milligram scale.

### 2.2. Sample Preparation

Sample preparation is a very important process in quality control of herbal medicine. The target compounds should be extracted prior to analysis. The extraction conditions for the major saponins in PJR including extraction solvent and extraction time were optimized by a univariate approach based on the total content of the four major saponins (expressed as peak area sum of the four saponins). The major saponins in PJR were triterpenoid saponins with several sugar units, including glucuronic acid, which are highly hydrophilic. Thus, different concentrations of aqueous methanol (50%, 70% and 90%, *v*/*v*) were tested and found that the highest content of the four major saponins could be achieved in the extract of 70% methanol (*v*/*v*). Ultra-sonication was used with 15 mL of 70% aqueous methanol (*v*/*v*) to extract 0.3 g of PJR powders for 30 min [15].

### 2.3. Optimization of the HPLC Conditions

All four major saponins contain a glucuronic acid unit, so a good separation and peak pattern will be achieved in the acidic mobile phase. It was found that a mobile phase consisting of 0.05% trifluoroacetic acid (*v*/*v*) (pH 2.3) is the most suitable mobile phase after testing formic acid, phosphoric acid, acetic acid, and trifluoroacetic acid with different concentrations. The chromatograms were monitored at UV 203 nm, which was widely adopted for the analysis of ginsenosides [2,11,13].

### 2.4. Method Validaton

Two different methods (M1 and M2) were developed due to different stock solution preparation methods. In M1, the stock solution was prepared by mixing the four pure analytes (CS-IV, CS-IVa, CS-V, PG-RT1) (P3C). In M2, the stock solution was directly prepared from the TPJS in which the four analytes were the major components. Next, the P3C and TPJS stock solutions were diluted to differently appropriate concentrations to produce calibration curves with 70% methanol (*v*/*v*), separately. Then, the linearity, regression, linear ranges, coefficient of determination (*R*^2^) values, intra- and inter-day variations, and LOD and LOQ of four analytes (CS-IV, CS-IVa, CS-V, PG-RT1) in P3C were determined using the developed HPLC method (M1, Table 1). The contents of CS-IV, CS-IVa, CS-V, and PG-RT1 in TPJS can be calculated from the calibration curve of the method M1 (Table 1). The contents of CS-V, PG-RT1, CS- IV, and CS-IVa in TPJS were found to be 29.10%, 8.43%, 19.57%, and 16.05%, respectively. An 8.01 mg of TPJS was dissolved in 1 mL of 70% methanol (*v*/*v*), which was used as the stock solution of TPJS. Thus, the concentration of CS-IV, CS-IVa, CS-V, and PG-RT1 in stock solution of TPJS were 2.33, 0.68, 1.57 and 1.29 mg/mL, respectively. The amount of each analytes in working solution of TPJS can also be calculated and those amounts were used for the determination of the linearity, regression, linear ranges, coefficient of determination (*R*^2^) values, intra- and inter-day variations, and LOD and LOQ of four analytes (CS-IV, CS-IVa, CS-V, PG-RT1) in TPJS with the same HPLC conditions (M2, Table 1 and Table 2). The coefficient of determination (*R*^2^ > 0.999) values of M2 indicates good correlations between the concentrations and peak areas of investigated saponins within the tested ranges. The overall LOD and LOQ of M2 were less than 1.5 and 6.0 μg/mL, respectively. The overall intra- and inter-day variations (RSD %) of the four analytes of M2 were less than 2.53% and 1.43%, respectively. For the repeatability test, the RSD of all analytes was less than 1.47% (Table 2), which indicates the method has good repeatability. The results of stability test shows the variation of analytes in solutions during the tested range is small (RSD ≤ 0.58%, Table 2), indicating that the sample and TPJS solutions were stable at room temperature (25 °C) for 24 h.

### 2.5. Quantification and Method Assessment

**Figure 2:**
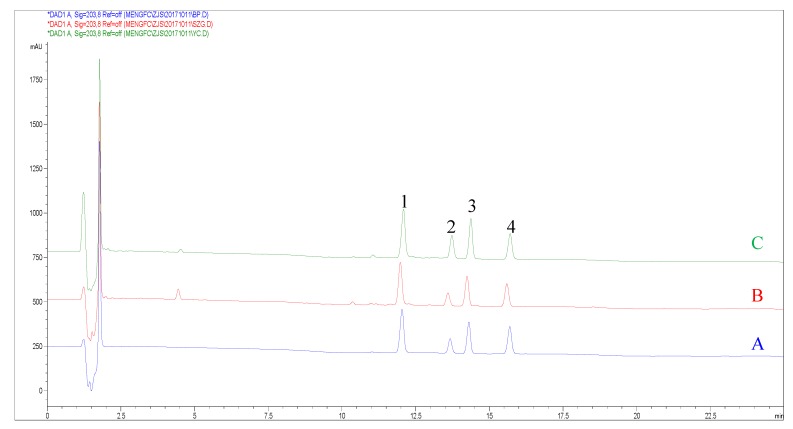
The HPLC chromatogram of (**A**) mixed standard references; (**B**) standardized reference fraction-TPJS; and (**C**) PJR-sample 1. 1: CS-V; 2: PG-RT1; 3: CS-IV; 4: CS-IVa.

Eleven samples of PJR from different locations were analyzed by the developed methods (M1 and M2). The identification of the investigated compounds was carried out in comparison of their retention time with the corresponding peak in mixed standards P3C and TPJS (Figure 2). As shown in the typical HPLC-UV chromatograms (Figure 2), the peak identification of each analyte is much more easily achieved than that of QAMS method using the relative retention time for peaks identification [15,23]. Another defect of QAMS method using UV detector is that all analytes should have similar maximum wavelength with the internal reference [24]. The developed method (M2) eliminated this shortcoming because all the analytes were analyzed according to their corresponding components in the standardized reference fraction by simply using multiple standard references (M1). The contents of the four analytes in PJR were calculated using the developed methods (M1 and M2). The results were summarized in Table 3. Percent difference (PD) was used to evaluate the method feasibility using the standardized reference fraction as the reference compounds for quantification of multiple analytes. The PD is calculated as: (|X − Y|)/[(X + Y)/2] × 100%. Here X and Y are the contents calculated by Methods 1 and 2. As shown in Table 3, the results from Methods 1 and 2 are very identical with that the PDs of all four analytes were all less than 2.11%. Thus, mEthod 2, using the standardized reference fraction as the reference compounds for quantification of multiple analytes, can be used for quality control of herbal medicine when multiple components need to be quantified.

## 3. Experimental

### 3.1. General

All analyses were performed on an Agilent 1200 series HPLC instrument (Palo Alto, CA, USA). Extraction was conducted on an Ultrasonic cleaner (Branson, MO, USA).

Chikusetsusaponins IV, IVa, V, and pseudoginsenoside RT1 were isolated from Panacis Japonici Rhizoma in previous research, the purity of each saponin was determined to be more than 98% by peak areas normalization analysis on HPLC [10,15].

Acetonitrile and methanol were purchased from Merck (Darmstadt, Germany). Trifluoroacetic acid was a product of Aladdin (Shanghai, China). Ultrapure water was purified by a Milli-Q purification system (Millipore, Bedford, MA, USA). D101 macroporous resin was a product of Haiguang (Tianjin, China). Ethanol for extraction and macroporous resin column chromatography was purchased from Kaitong (Tianjin, China). Millex-FG PTFE syringe-driven filters (filter diameter 13 mm, pore size 0.20 μm) were purchased from Millipore (Cork, Ireland).

PJR samples were collected or purchased from different locations of China, which were authenticated by one of the authors, Professor Ping Chen. The voucher specimens were deposited at the College of Biology and Pharmaceutical engineering, Wuhan Polytechnic University, Wuhan, China.

### 3.2. Preparation of Total P. japonicus Saponin

Dried PJR (1 kg) was powdered and extracted with 95% ethanol (*v*/*v*) (3 × 5 L) under reflux. The ethanol extract was concentrated under vacuum to remove ethanol and then suspended in water. The water solution was loaded on a D 101 macroporous resin (1.0 kg) column and eluted by water, 20% ethanol-water (*v*/*v*) and 80% ethanol-water (*v*/*v*) (each 8 L). The 80% ethanol-water fraction was pooled and dried in vacuum to afford 106.5 g of the total *P. japonicus* saponin (TPJS).

### 3.3. Preparation of Standard Solutions

The mixed four saponins (CS-V 1.02 mg; PG-RT1 1.01 mg; CS-IV 1.03 mg and CS-IVa 1.04 mg) were dissolved in 1 mL of 70% methanol (*v*/*v*) and stored in 4 °C, which was served as P3C stock solution. The TPJS stock solution was prepared from an 8.01 mg of TPJS dissolved in 1 mL of 70% methanol (*v*/*v*) and stored at 4 °C. Then the P3C and TPJS stock solutions were diluted to differently appropriate concentrations to produce calibration curves with 70% methanol (*v*/*v*), separately.

### 3.4. Preparation of Sample Solutions

About 0.3 g of powdered (80 mesh) PJR was accurately weighed and then sonicated with the optimized condition in previous research (30 min, 15 mL of 70% methanol, *v*/*v*) in a conical flask [15]. The sample was cooled to room temperature and then was made up to its original weight with a specific amount of 70% methanol (*v*/*v*). The extraction was filtered through a syringe filter (0.20 μm), and the subsequent filtrate was stored in 4 °C as the sample solution for analysis. Sample 1 was used as the sample for extraction conditions, precision, stability, and recovery test.

### 3.5. HPLC Conditions

All analyses were performed on an Agilent 1200 series HPLC instrument, and an Agilent Eclipse XDB-C18 column (4.6 mm × 150 mm, 5 μm) was used. The mobile phase consisted of water containing 0.05% trifluoroacetic acid (pH 2.3) (A) and acetonitrile (B) with the following gradient program: 0–5 min, 25–30% B; 5–7 min, 30–35% B; 7–10 min, 35% B; 10–12 min, 35–37% B; 12–15 min, 37% B; 15–17 min, 37–40% B; 17–22 min, 40% B; 22–25 min, 40–50% B. The injection volume was 20 μL. The flow rate was 1.0 mL/min. The column temperature was maintained at 30 °C. The saponins were monitored at 203 nm.

### 3.6. Calibration Curves, and Limits of Detection and Quantification

The P3C working solution containing four reference compounds (CS-IV, CS-IVa, CS-V, PG-RT1) with nine different concentrations were analyzed in triplicate using the HPLC method described above. The calibration curves were constructed by plotting the mean peak areas vs. the concentration of each analyte. The quantitation of each analyte in TPJS and the samples were performed based on their individual calibration curve (Method 1). Similarly, the working solution of TPJS with nine different concentrations were analyzed in triplicate using the same HPLC method to construct the calibration curves, which was used for quantification of four saponins (CS-IV, CS-IVa, CS-V, PG-RT1) in PJR (Method 2).

The working solutions (P3C and TPJS) with the lowest concentration were diluted with 70% methanol (*v*/*v*) to yield a series of appropriate concentrations. The limits of detection (LOD) and quantification (LOQ) were separately determined in triplicate at a signal-to-noise ratio (S/N) of 3 and 10, respectively, by comparing measured signals from samples with known low concentrations of analyte with those of blank samples. 

### 3.7. Precision, Repeatability, Stability, and Accuracy

The precision of the developed method was evaluated by measurements of intra- and inter-day variability. For the intra-day variability test, the mixed reference solutions and the TPJS solution were analyzed to obtain six replicates within one day. For the inter-day variability test, the solutions were examined in duplicate for three consecutive days. Quantities of the analytes were calculated from their corresponding calibration curves. The relative standard deviation (RSD) was used to evaluate precision.

The Sample 1 solution was stored in an injection vial at 25 °C. The analyses were performed at 0, 2, 4, 8, 12, and 24 h, respectively. The RSD was used to evaluate the stability.

A spiked recovery test was used to evaluate the accuracy of the method. Known amounts of the four reference compounds or TPJS were added to approximate 0.1 g of Sample 1 powder, and then extracted and analyzed as described above. The recovery was calculated with the following equation: Recovery (%) = (amount determined − amount original)/amount spiked × 100%. The accuracy was evaluated by calculating the mean recoveries.

## 4. Conclusions

A novel strategy using standardized reference fraction for qualification of the major saponins in Panacis Japonici Rhizoma was developed to easily and effectively control the quality of PJR. The strategy is feasible and reliable, and the methodology is also validated based on the developed method. The standardized reference fraction is used for quantification, which might solve the issues arising from the shortage of the pure reference compounds in quality control of herbal medicines.

## Figures and Tables

**Table 1:** Calibration curves, linear range, LOD, and LOQ of the investigated compounds in Methods 1 and 2.

Method	Analytes	Regression Equation	*R*²	Linear Range (mg/mL)	LOD (μg/mL)	LOQ (μg/mL)
M1	CS-V	*y* = 5026.2*x* + 15.32	0.9998	0.004–1.025	0.7	2.9
	PG-RT_1_	*y* = 5303.1*x* + 30.28	0.9998	0.004–1.010	0.5	1.9
	CS-IV	*y* = 5157.5*x* + 30.64	0.9996	0.004–1.030	1.0	3.9
	CS-IVa	*y* = 5140*x* + 14.70	0.9998	0.004–1.045	0.8	3.3
M2	CS-V	*y* = 5193.6*x* + 42.02	0.9993	0.036–1.166	1.5	6.0
	PG-RT_1_	*y* = 5233.9*x* + 34.39	0.9992	0.011–0.676	0.5	1.9
	CS-IV	*y* = 5301.8*x* + 11.44	0.9999	0.024–0.793	1.0	3.9
	CS-IVa	*y* = 5020*x* + 47.29	0.9993	0.020–1.286	0.7	2.8

**Table 2:** The accuracy, precision, repeatability, and stability of Methods 1 and 2.

Method	Analytes	Recovery (%, RSD, *n* = 6)	Precision (%, RSD, *n* = 6) Intra-Day Inter-Day		Repeatability (%, RSD, *n* = 6)	Stability (%, RSD, *n* = 6)
M1	CS-V	100.70, 1.11	1.69	0.95	0.94	0.58
	PG-RT_1_	98.15, 1.36	1.41	1.06	0.28	0.49
	CS-IV	102.26, 1.12	1.94	1.43	1.04	0.48
	CS-IVa	100.29, 2.35	2.53	1.06	1.47	0.54
M2	CS-V	98.66, 0.48	0.23	0.12	0.18	0.58
	PG-RT_1_	100.85, 0.69	0.72	1.33	0.57	0.49
	CS-IV	96.65, 0.41	0.40	0.55	0.34	0.48
	CS-IVa	103.33, 0.81	0.30	0.71	0.30	0.54

**Table 3:** Comparison of the contents (%) in PJR calculated by individual (M1) and TPJS cailbration curves (M2).

No.	CS-V			PG-RT1			CS-IV			CS-IVa		
	**M1**	**M2**	**PD %**	**M1**	**M2**	**PD %**	**M1**	**M2**	**PD %**	**M1**	**M2**	**PD %**
1	6.40	6.35	0.78	2.88	2.91	1.03	4.85	4.91	1.23	3.34	3.39	1.49
2	5.27	5.21	1.15	3.12	3.17	1.59	4.08	4.13	1.22	1.48	1.49	0.67
3	8.92	8.91	0.11	2.45	2.48	1.21	5.22	5.30	1.52	4.57	4.65	1.74
4	11.33	11.35	0.18	2.21	2.24	1.34	1.87	1.87	0.00	7.52	7.68	2.11
5	8.74	8.72	0.22	3.87	3.93	1.54	4.03	4.07	0.99	7.07	7.22	2.10
6	5.81	5.76	0.86	0.94	0.95	1.05	2.56	2.57	0.39	3.14	3.18	1.27
7	4.19	4.12	1.70	2.17	2.19	0.91	4.42	4.48	1.35	2.47	2.49	0.81
8	8.41	8.40	0.12	2.66	2.70	0.0149	4.47	4.52	1.11	5.43	5.53	1.82
9	8.59	8.57	0.23	0.27	0.27	0.00	7.52	7.65	1.01	1.50	1.50	0.00
10	11.28	11.31	0.27	2.37	2.40	1.26	6.04	6.13	1.48	4.55	4.63	1.74
11	8.77	8.76	0.11	0.63	0.62	1.60	5.09	5.16	1.37	1.75	1.75	0.00
